# Association between body roundness index and female infertility: a cross-sectional study of NHANES 2013–2018

**DOI:** 10.3389/fnut.2024.1509311

**Published:** 2024-12-17

**Authors:** Haiyan Li, Zhenbo OuYang, Ziyao Ding, Xianyue Hu, Yanjing Bao, Tianyang Gao, Wenfeng Hua

**Affiliations:** ^1^Department of Reproductive Medicine Center, The Affiliated Guangdong Second Provincial General Hospital of Jinan University, Guangzhou, Guangdong, China; ^2^Department of Gynecology, The Affiliated Guangdong Second Provincial General Hospital of Jinan University, Guangzhou, Guangdong, China; ^3^First Clinical Medical College, Xuzhou Medical University, Xuzhou, Jiangsu, China; ^4^The Second School of Clinical Medicine, Southern Medical University, Guangzhou, Guangdong, China; ^5^Research Institute for Maternal and Child Health, The Affiliated Guangdong Second Provincial General Hospital of Jinan University, Guangzhou, Guangdong, China

**Keywords:** visceral obesity, female infertility, body roundness index, waist circumference, NHANES

## Abstract

**Background:**

The use of visceral obesity as an indicator for predicting female infertility risk has not been well established. The body roundness index (BRI) is a novel, non-invasive indicator of visceral fat; however, previous reports have not addressed the relationship between the BRI and female infertility. This study sought to fill this research gap by investigating the association between the BRI and the risk of female infertility.

**Methods:**

This cross-sectional study examined 3,528 women aged 18 to 45 who participated in the National Health and Nutrition Examination Survey (NHANES) from 2013 to 2018. Infertility was defined based on responses to the reproductive health questionnaire. The BRI was calculated using waist circumference and height. Covariates included demographic traits, physical exam results, laboratory test findings, and survey data. Weighted multivariable logistic regression models and spline smoothing analysis assessed the relationship between the BRI and infertility. Bayesian statistics were used to examine the robustness of significant associations.

**Results:**

Based on their self-report data, 407 (11.54%) participants were classified as having infertility. A significantly higher percentage of participants with a higher BRI were found to have infertility. Multivariable logistic regression revealed that the BRI was significantly associated with increased female infertility risk, regardless of independent variable analysis by continuous variable or quartile (Q1 to Q4) in the fully adjusted model (Model 3, continuous variable: OR = 1.1, 95% confidence interval [CI] = 1.05–1.16, *p* = 0.0009; Q4 vs. Q1: OR = 2.16, 95% CI = 1.38–3.39, *p* = 0.0035, *P*_trend_ = 0.004). Non-linear and threshold effects in the relationship between the BRI and female infertility were identified, with an inflection point of 6.36. Subgroup analyses showed that this positive association remained consistent across most demographic and health-related categories. The Bayesian statistics analyses further confirmed the robustness of these findings.

**Conclusion:**

A positive non-linear relationship exists between the BRI and the risk of female infertility, suggesting that the BRI could serve as a valuable indicator in female fertility assessments.

## Introduction

Infertility is a universal health issue, affecting approximately 10% of reproductive-aged couples attempting to conceive. It is defined as the failure to establish a clinical pregnancy after 1 year of regular, unprotected sexual intercourse (1, 2). The World Health Organization has categorized infertility as a societal disorder, and the U.S. Centers for Disease Control and Prevention (CDC) has designated it a public health priority. Therefore, identifying potential risk factors and reliable markers for the prevention and management of infertility holds significant public health importance (3–5).

Obesity is a major health challenge because it substantially increases the risk of cardiovascular and cerebrovascular diseases, metabolic disorders, and cancers, as well as infertility (6, 7). Obesity, defined as a body mass index (BMI) of ≥30 kg/m^2^, is characterized by long-term metabolic disorders, excessive fat accumulation, and metabolic alterations. As the most commonly utilized body mass indicator, the BMI has been used to diagnose various disorders associated with overweight and obesity. However, the BMI does not distinguish between subcutaneous and visceral fat, making its use controversial. Compared to the subcutaneous fat that lies just under the skin around the belly, the visceral fat that wraps around internal organs is strongly linked to metabolic diseases, insulin resistance, and an increased risk of death, even in individuals with a normal BMI (8, 9). A similar controversy regarding the BMI has been reported about male infertility (10). For instance, Bian et al. discovered that among couples undergoing infertility treatment, even in men with normal BMI, a higher male waist circumference (WC) was associated with a lower sperm concentration and lower probability of achieving a live birth (11). Additionally, the BMI cannot distinguish between muscle and fat mass. Therefore, to address the limitations of BMI, it is essential to use a novel indicator to evaluate visceral fat to accurately comprehend the effects of obesity on fertility.

The body roundness index (BRI) is a novel obesity-related anthropometric index that more accurately reflects body fat based on human body shape than other existing measures. It is used to calculate both body fat and total visceral fat percentages (12). Recently, many studies have reported that the BRI is an independent risk factor associated with all-cause mortality (13), hypertension (14), colorectal cancer (15), cardiovascular disease (16, 17), and osteoporosis (18). Notably, Zhang et al. found a significant positive correlation between the BRI and depression, showing that for each unit increase in the BRI, the prevalence of depression increased by 8% (19). Additionally, Li et al. observed that higher baseline BRI levels are linked to the development of metabolic syndrome (MetS). Baseline BRI may help identify patients at risk for MetS, which can lead to early and optimal treatment to improve patient outcomes (20). Furthermore, the risk of developing diabetes and prediabetes increased by 17% for each unit increase in the BRI, even after adjusting for other factors (21). Depression, MetS, and diabetes are closely linked to female infertility. However, the association between the BRI and infertility remains unexplored. To address this gap, this study aims to examine the association between the BRI and female infertility by utilizing data from the National Health and Nutrition Examination Survey (NHANES) and to explore the potential of the BRI as an independent predictor of infertility.

## Methods

### Data source and study population

The NHANES database used in this study has been made publicly available by the National Center for Health Statistics (NCHS), a division of the Centers for Disease Control and Prevention (CDC). The NHANES is a series of nationally representative and cohort surveys designed to monitor and assess the nutritional and health status of the U.S. population. Since 1999, the NHANES has conducted a biennial survey cycle, conducting in-home interviews to gather demographic and health information while conducting physical examinations and laboratory tests at a mobile examination center to collect biological samples and clinical data. The NCHS Ethics Review Board granted authorization for the participation of human subjects in the NHANES, and all participants provided written informed consent for the collection of their data.

**Figure 1:**
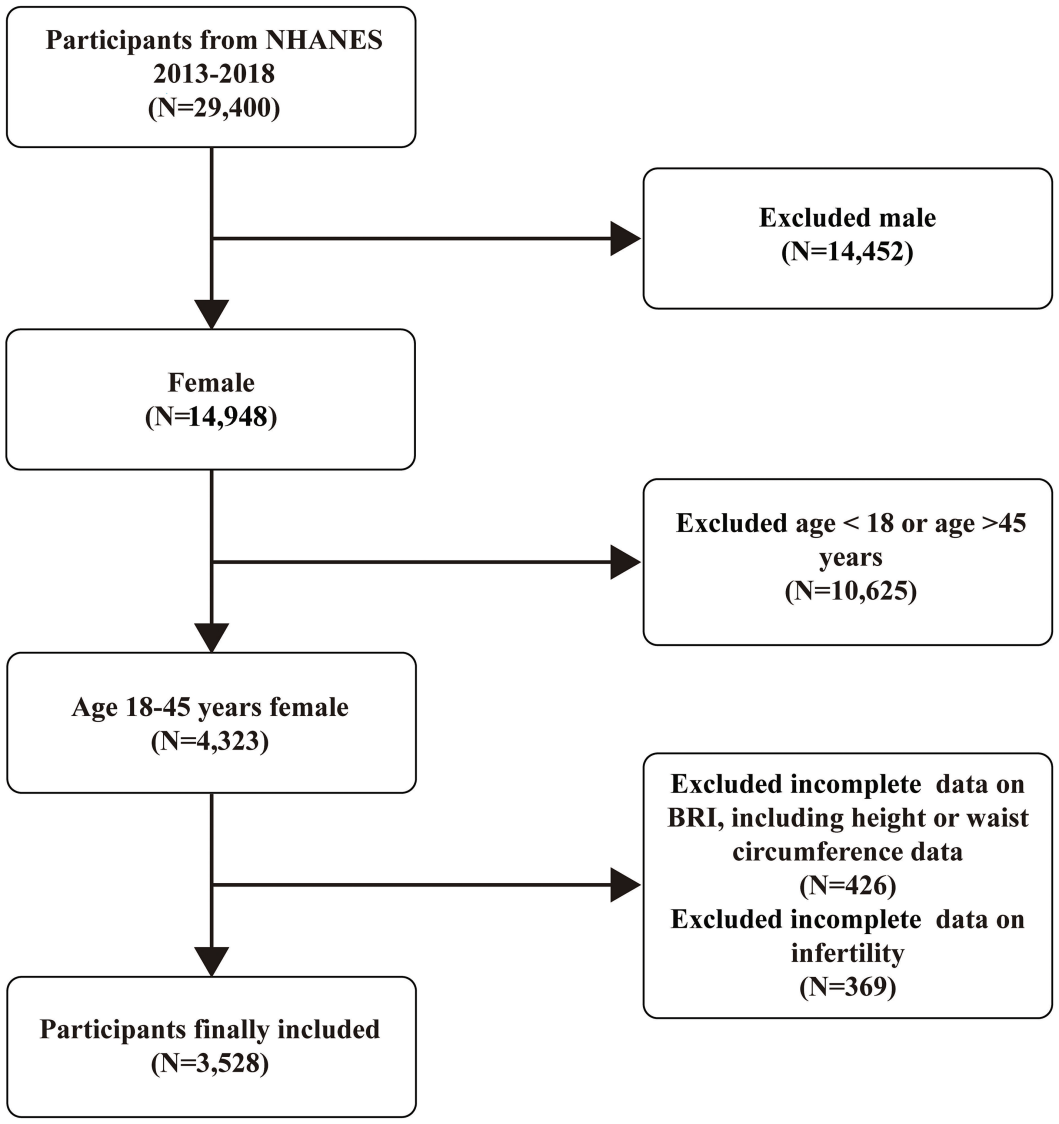
Inclusion and exclusion criteria flowchart.

Participants from 2013 to 2018, which constituted three cycles of the NHANES, were included in the study. All participants were non-pregnant women aged 18 to 45 years, representative of the non-institutionalized civilian resident U.S. population. An initial sample of 29,400 participants provided comprehensive information on their BRI and infertility status. After excluding male participants (*n* = 14,452), female participants aged above 45 or below 18 years (*n* = 10,625), female participants without BRI data (*n* = 426), and female participants without infertility information (*n* = 369), the final sample included 3,528 eligible participants (Figure 1).

### Calculation of BRI

The BRI was calculated using a formula used in previous studies (12), and the height and WC measurements were obtained at mobile examination centers. The formula is as follows:


$$BRI=364.2–365.5\times \surd \left[1–{\left(WC/\left(2\pi \right)\right)}^{2}/{\left(0.5\times \mathrm{height}\right)}^{2}\right]$$

### Definition of infertility

Infertility, the dependent variable, was measured by asking the following questions from the Reproductive Health Questionnaire: “Have you ever attempted to become pregnant for at least 1 year without success?” (RHQ074) and “Have you ever consulted a doctor or other medical provider due to an inability to become pregnant?” (RHQ076). Participants who responded “yes” to either question were categorized as having infertility; those who responded “no” were categorized as not having infertility.

### Covariates

Covariates in this study included age, ethnicity, marital status, educational level, poverty income ratio (PIR), alcohol consumption status, hypertension (yes/no), diabetes (yes/no), dyslipidemia (yes/no), age at menarche, history of pelvic infection/pelvic inflammatory disease (PID) (yes/no), use of birth control pills (yes/no), use of female hormones (yes/no), height, WC, BMI, and blood cotinine levels. Detailed information on the procedures for obtaining these covariates is available on the NHANES official website.

### Statistical analysis

All statistical analyses were conducted while considering the intricate, multistage clustered nature of the surveys and using suitable NHANES sampling weights, following CDC guidelines. In the descriptive analyses, the differences in baseline characteristics between the participants were grouped by infertility status and BRI quartile. Means with 95% confidence intervals (CIs) were used to present continuous variables, while percentages with 95% CIs were used to present categorical variables. The study population’s baseline characteristics were evaluated using a weighted linear regression model and weighted chi-square test. For missing data, continuous variables were imputed using medians or means based on the data distribution, and categorical variables were imputed using the modes. The proportion of missing data varied across different variables: BMI (0.09%), marital status (10.97%), PIR (8.14%), diabetes (54.06%), blood cotinine (4.71%), drinking (2.86%), menarche (0.51%), PID (0.54%), birth control pills (0.09%), and female hormones (11.14%).

Subsequently, weighted multivariate logistic regression models were used that included known or potential confounders to investigate the association between the BRI and infertility risk. To explore non-linear relationships, smooth curve fitting and threshold effect analysis were performed, a recursive algorithm was used to identify inflection points, and a two-segment linear regression model was applied on either side of the inflection point (K). Briefly, the K value was determined using a two-step recursive method. Step 1 is to narrow the value to a 10-percentile range of the independent variable. From 5 to 95%, incremented by 5% to find out which percentile points give the model the highest likelihood; step 2 is to refine this range using quartiles and recursive narrowing until the precise K value yielding the maximum likelihood segmented regression model is identified.

Subgroup analyses were performed to investigate the relationship between the BRI and infertility across different subgroups including age, BMI, ethnicity, and the status of hypertension, dyslipidemia, and diabetes. Finally, the false positive report probability (FPRP) and Bayesian false discovery probability (BFDP) tests were used to further evaluate the robustness of the significant findings, which were described in detail in our previous study (22). The FPRP evaluates the likelihood of no true association between the BRI and the risk of female infertility. This assessment is affected by statistical power, the observed *p*-value, and prior probability. Using SAS software, we calculated the statistical power and FPRP values with an odds ratio (OR) of 1.50 for risk and 0.67 for protective effects, across prior probabilities ranging from 0.25 to 0.01. We applied a cutoff value of 0.2, as previously recommended, which serves as the threshold for FPRP; values below 0.2 are considered significant (23). Additionally, the BFDP was used to assess the significance of the results, taking into account the cost of false discoveries and non-discoveries. The cutoff value for BFDP was set at 0.8, assuming that a false non-discovery is four times more costly than a false discovery. The same prior probabilities used for FPRP were applied to BFDP, with values less than 0.8 regarded as noteworthy (24).

The statistical analyses for this study were performed using R, EmpowerStats, and SAS 9.4 software (SAS Institute Inc., Cary, NC, United States). A *p*-value of <0.05 was considered statistically significant.

## Results

### Participant characteristics

Weighted analysis of the participants in this study revealed that the BRI values in the infertility group were significantly higher than those of the non-infertility group (Supplementary Table S1). This result suggests the potential of the BRI as a risk predictor for female infertility. As shown in Table 1, grouping participants by BRI quartiles revealed significant differences in various characteristics, including age, ethnicity, marital status, education level, BMI, PIR, alcohol consumption, height, WC, diabetes, dyslipidemia, hypertension, age at menarche, history of PID, use of birth control pills, use of female hormones, and infertility status (*p* < 0.05), but not in blood cotinine level. Notably, the mean WC showed a steady increase from 75.55 cm (Q1) to 120.64 cm (Q4) across the quartile population. Similarly, the percentage of participants experiencing infertility increased significantly from Q1 to Q4 (*p* < 0.0001) and was notably higher in Q3 and Q4 (14.74 and 18.49%, respectively) than in Q1 and Q2 (7.18 and 11.57%, respectively). These differences suggest that the potential links between the BRI and infertility warrant further research.

**Table 1:** Basic characteristics of study population based on BRI quartiles^a^.

Variables	Q1 (1.25–3.09)	Q2 (3.09–4.18)	Q3 (4.18–5.53)	Q4 (5.53–23.48)	*p*
**Age (years)**	28.38 (27.69–29.06)	31.51 (30.86–32.16)	33.09 (32.28–33.89)	33.04 (32.46–33.61)	<0.0001
**Ethnicity (%)**					<0.0001
Mexican American	5.60 (3.94–7.90)	10.32 (7.91–13.35)	16.68 (12.83–21.39)	15.91 (12.19–20.49)	
Other Hispanic	7.74 (5.86–10.16)	8.30 (5.91–11.54)	8.53 (6.36–11.35)	7.04 (5.56–8.87)	
Non-Hispanic White	63.26 (57.74–68.46)	57.65 (51.64–63.44)	49.42 (43.05–55.81)	53.09 (47.08–59.02)	
Non-Hispanic Black	11.05 (8.56–14.15)	11.13 (8.95–13.75)	14.47 (10.95–18.87)	17.18 (13.34–21.85)	
Other ethnicities- Including multi-racial	12.35 (10.03–15.12)	12.60 (10.19–15.47)	10.91 (8.31–14.20)	6.78 (5.30–8.63)	
**Marital status (%)**					<0.0001
Married	35.07 (31.20–39.13)	47.18 (42.79–51.62)	45.54 (40.24–50.94)	44.20 (39.97–48.52)	
Widowed	0.07 (0.01–0.53)	0.23 (0.08–0.65)	1.36 (0.51–3.58)	0.43 (0.16–1.14)	
Divorced	3.27 (2.12–5.00)	6.96 (4.87–9.84)	8.69 (6.42–11.66)	6.53 (4.71–8.98)	
Separated	1.18 (0.64–2.18)	2.49 (1.57–3.91)	4.06 (2.91–5.63)	3.88 (2.59–5.76)	
Never married	48.86 (44.59–53.15)	28.79 (25.34–32.51)	25.40 (21.22–30.08)	29.43 (25.36–33.86)	
Living with partner	11.55 (8.94–14.78)	14.35 (11.70–17.49)	14.95 (11.99–18.50)	15.54 (12.93–18.56)	
**Education level (%)**					0.0002
Less than high school	10.14 (7.93–12.88)	10.66 (8.52–13.26)	14.52 (11.93–17.55)	15.91 (13.05–19.25)	
High school or equivalent	18.52 (15.34–22.19)	19.74 (16.30–23.69)	21.97 (18.06–26.45)	23.35 (20.34–26.65)	
College or above	71.34 (66.47–75.77)	69.60 (64.74–74.06)	63.51 (58.78–68.00)	60.75 (55.85–65.44)	
**BMI (kg/cm** ^**2**^ **)**	21.08 (20.90–21.26)	25.43 (25.20–25.65)	30.72 (30.44–30.99)	40.03 (39.51–40.56)	<0.0001
**PIR**	2.87 (2.73–3.02)	2.84 (2.68–3.01)	2.44 (2.29–2.59)	2.24 (2.06–2.42)	<0.0001
**Blood cotinine (ng/mL)**	46.52 (36.63–56.41)	36.13 (28.41–43.84)	42.38 (34.86–49.90)	42.50 (33.28–51.72)	0.3364
**Drinking (%)**					<0.0001
Non-drinker	29.48 (25.03–34.37)	33.83 (29.00–39.01)	38.19 (33.76–42.82)	43.52 (38.86–48.29)	
1–5 drinks/month	31.74 (27.88–35.86)	30.72 (26.76–34.98)	34.04 (30.28–38.02)	36.39 (32.46–40.52)	
5–10 drinks/month	17.27 (13.26–22.18)	15.21 (12.63–18.21)	14.42 (11.36–18.13)	11.08 (8.66–14.08)	
10^+^ drinks/month	21.51 (17.26–26.47)	20.24 (16.38–24.75)	13.35 (10.58–16.70)	9.01 (6.64–12.12)	
**Height (cm)**	163.77 (163.13–164.42)	162.60 (161.96–163.25)	162.17 (161.56–162.79)	161.94 (161.39–162.48)	0.0018
**WC (cm)**	75.55 (75.03–76.08)	87.07 (86.67–87.47)	99.53 (98.97–100.09)	120.64 (119.34–121.93)	<0.0001
**Diabetes (%)**					<0.0001
Yes	0.11 (0.01–0.79)	1.04 (0.51–2.09)	2.95 (1.92–4.53)	7.84 (6.13–9.98)	
No	99.89 (99.21–99.99)	98.96 (97.91–99.49)	97.05 (95.47–98.08)	92.16 (90.02–93.87)	
**Dyslipidemia (%)**					<0.0001
Yes	9.03 (6.19–13.00)	13.17 (10.79–15.99)	19.37 (15.90–23.39)	23.59 (19.91–27.73)	
No	90.97 (87.00–93.81)	86.83 (84.01–89.21)	80.63 (76.61–84.10)	76.41 (72.27–80.09)	
**Hypertension (%)**					<0.0001
Yes	4.66 (3.14–6.87)	10.89 (9.05–13.06)	15.50 (12.90–18.50)	27.53 (23.61–31.83)	
No	95.34 (93.13–96.86)	89.11 (86.94–90.95)	84.50 (81.50–87.10)	72.47 (68.17–76.39)	
**Menarche (years)**	12.98 (12.82–13.15)	12.69 (12.58–12.81)	12.40 (12.23–12.57)	12.18 (12.03–12.33)	<0.0001
**PID (%)**					0.0200
Yes	3.29 (2.19–4.90)	3.37 (2.24–5.05)	5.23 (3.53–7.67)	6.48 (4.39–9.48)	
No	96.71 (95.10–97.81)	96.63 (94.95–97.76)	94.77 (92.33–96.47)	93.52 (90.52–95.61)	
**Birth control pills (%)**					0.6238
Yes	70.38 (66.22–74.22)	72.45 (68.03–76.47)	73.38 (69.80–76.67)	70.83 (66.49–74.82)	
No	29.62 (25.78–33.78)	27.55 (23.53–31.97)	26.62 (23.33–30.20)	29.17 (25.18–33.51)	
**Female hormones (%)**					0.0012
Yes	1.65 (0.83–3.24)	4.97 (3.27–7.49)	6.35 (4.12–9.66)	4.42 (2.93–6.62)	
No	98.35 (96.76–99.17)	95.03 (92.51–96.73)	93.65 (90.34–95.88)	95.58 (93.38–97.07)	
**Infertility (%)**					<0.0001
Yes	7.18 (5.32–9.62)	11.57 (8.73–15.19)	14.74 (12.25–17.65)	18.49 (14.79–22.87)	
No	92.82 (90.38–94.68)	88.43 (84.81–91.27)	85.26 (82.35–87.75)	81.51 (77.13–85.21)	

BMI, body mass index; PIR, poverty impact ratio; WC, waist circumference; PID, pelvic infection/pelvic inflammatory disease; BRI, body roundness index.

a Percentage estimates were nationally representative through the use of survey weights.

### Association between BRI and infertility

The correlation between BRI and infertility is presented in Table 2. Logistic regression model analysis revealed a significantly positive correlation between the BRI and infertility. When the BRI was used as a continuous variable, the odds ratio (OR) of Model 1 (unadjusted) was 1.13 (95% CI = 1.08–1.19, *p* < 0.0001). After adjusting the age and ethnicity (Model 2) and in the fully adjusted model (Model 3), the OR values decreased slightly to 1.11 (95% CI = 1.05–1.17, *p* = 0.0004) and 1.11 (95% CI = 1.05–1.16, *p* = 0.0009), respectively. Further analysis using the BRI quartiles supported this finding, showing that the highest quartile of the BRI (Q4) was significantly associated with increased infertility risk (OR = 2.16, 95% CI = 1.38–3.39, *p* = 0.0035) compared to the lowest quartile (Q1) in Model 3. Trend analyses also demonstrated that women with a higher BRI had a significantly elevated risk of infertility (*p* = 0.004).

**Table 2:** Association between the BRI and female infertility.

Exposures	Model 1	Model 2	Model 3
	OR (95% CI), *p*	OR (95% CI), *p*	OR (95% CI), *p*
**BRI (continuous)**	1.13 (1.08–1.19), <0.0001	1.11 (1.05–1.17),0.0004	1.11 (1.05–1.16), 0.0009
BRI (quartile)			
Q1	Reference	Reference	Reference
Q2	1.69 (1.11–2.58), 0.0186	1.45 (0.95–2.22), 0.0944	1.37 (0.91–2.07), 0.1535
Q3	2.24 (1.53–3.27), 0.0002	1.81 (1.22–2.67), 0.0052	1.63 (1.07–2.48), 0.0360
Q4	2.93 (1.94–4.44), <0.0001	2.38 (1.52–3.73), 0.0005	2.16 (1.38–3.39), 0.0035
***P* for trend**	<0.0001	0.0010	0.0040

Model 1: Non-adjusted.

Model 2: Adjusted for age and ethnicity.

Model 3: Further adjusted for education level, PIR, marital status, drinking, blood cotinine, dyslipidemia, diabetes, hypertension, menstrual status, PID, birth control pills, and female hormones.

### Non-linear relationship between the BRI and infertility

**Figure 2:**
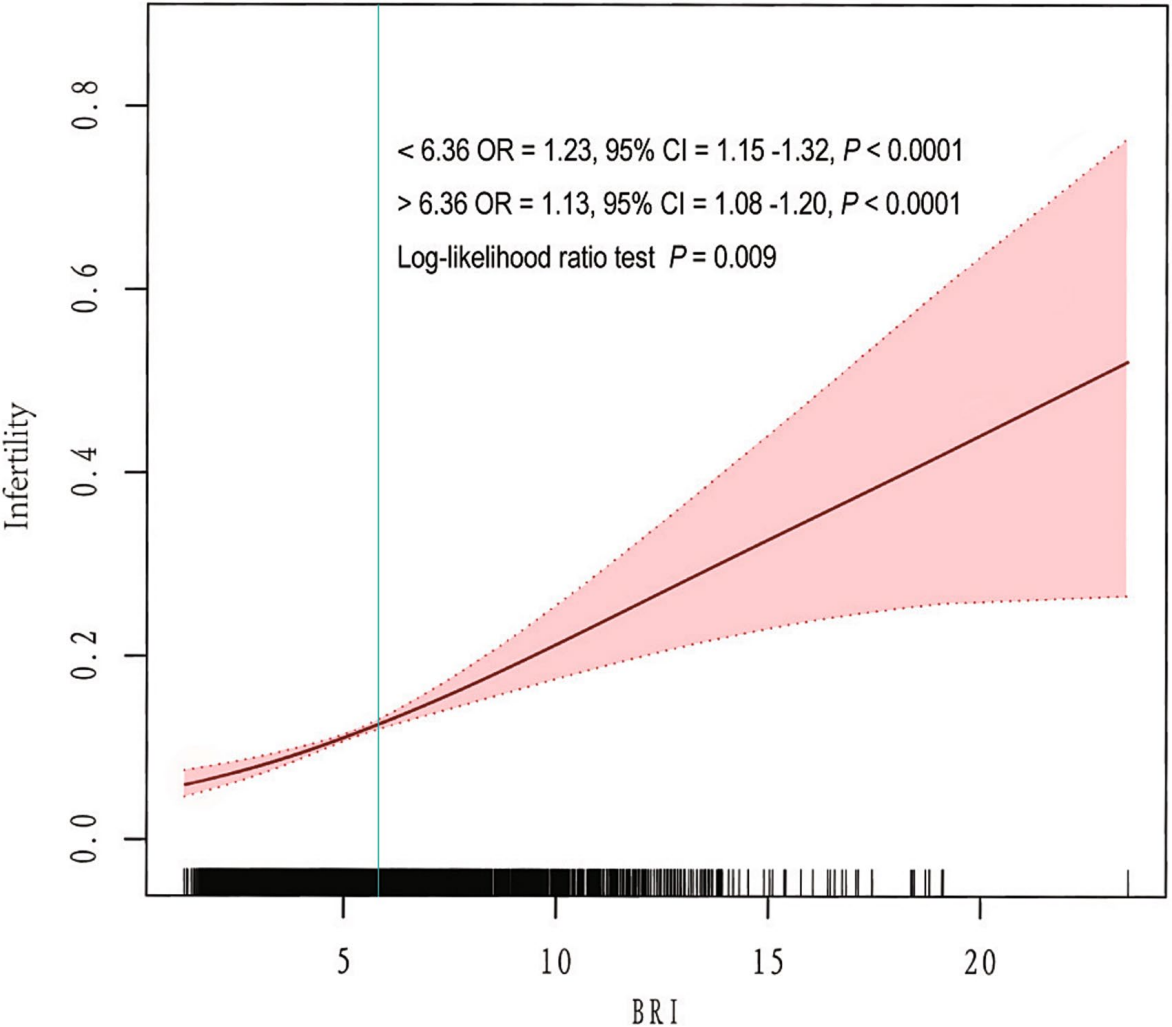
Non-linear relationship between the BRI and female infertility.

To better understand the relationship between the BRI and infertility, a spline smoothing analysis was performed using the generalized additive model. The analysis revealed a non-linear positive relationship between the BRI and infertility (Figure 2). To further evaluate this relationship in detail, a threshold effect analysis was performed using a weighted two-segment linear regression model and a recursive algorithm. The calculated inflection point was found to be 6.36, with a log-likelihood ratio test *p*-value of 0.009. Below the BRI threshold of 6.36, each unit increase in the BRI was associated with a 1.23-fold increase in the risk of infertility (OR = 1.23, 95% CI = 1.15–1.32, *p* < 0.0001). Above this threshold, each unit increase in the BRI corresponded to a 1.13-fold increase in the risk of infertility (OR = 1.13, 95% CI = 1.08–1.20, *p* < 0.0001; Table 3).

**Table 3:** Threshold effect analysis of the BRI and female infertility.

Infertility	OR (95% CI)	*p*
BRI		
Model I	1.15 (1.10–1.21)	<0.0001
Model II		
**Inflection point (K)**	6.36	
<K point effect 1	1.23 (1.15–1.32)	<0.0001
>K point effect 2	1.13 (1.08–1.20)	<0.0001
Effect 2 minus effect1	0.92 (0.87–0.98)	0.0088
Predicted value of the equation at the folding point	−1.81 (−1.89–1.73)	
Log-likelihood ratio test		0.009

Adjusted for education level, PIR, marital status, drinking, blood cotinine, dyslipidemia, diabetes, hypertension, menstrual status, PID, birth control pills, and female hormones.

### Subgroup analysis

**Figure 3:**
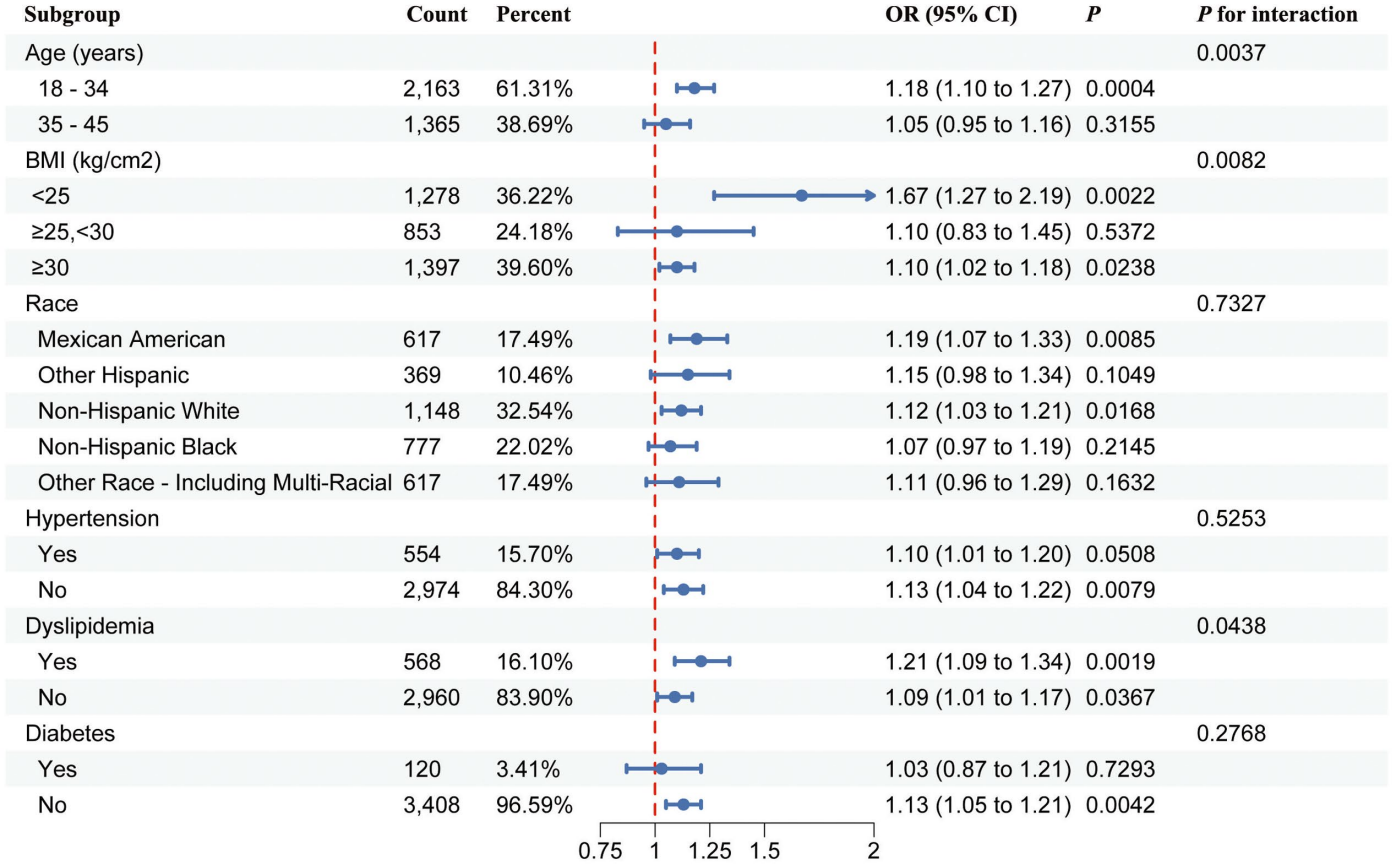
Forest plot of stratified analysis and interaction effects for the association between the BRI and female infertility.

Subgroup analysis and interaction tests were performed to explore the strength of the correlation between the BRI and infertility across different populations. The results showed that the relationship between the BRI and infertility was influenced by factors such as age, ethnicity, BMI, hypertension, dyslipidemia, and diabetes status (Figure 3). Moreover, significant interaction effects were observed in the subgroups based on age, BMI, and dyslipidemia subgroups (*P* for interaction <0.05) in Model 3, suggesting that these covariates interacted with the BRI to influence infertility in different ways (Figure 3).

### FPRP and BFDP values for all significant associations

To examine the statistical robustness of the significant associations and determine whether the findings warranted further analysis, FPRP and BFDP analyses were conducted. As shown in Table 4, the thresholds for the FPRP and BFDP were defined as 0.2 and 0.8, respectively. The analysis showed that all significant associations were noteworthy at a prior probability level of 0.25. When a prior probability of 0.1 was assumed, all significant associations were noteworthy for both tests, except for Q2 in the FPRP test. Moreover, at a prior probability level of 0.01, all significant associations remained noteworthy for both tests, except those for Q2. Notably, when assuming a prior probability of 0.001, the significant association of Q4 remained noteworthy in the BFDP test (OR = 2.93, 95% CI = 1.94–4.44, *p* < 0.0001, BFDP value = 0.726). However, at a prior probability of 0.0001, no noteworthy results were observed for any significant association in either the FPRP or BFDP tests.

**Table 4:** FPRP and BFDP analyses for significant findings.

	Crude OR^a^ (95% CI)	*p*^b^	Statistical power^c^	Prior probability FPRP/BFDP^d^				
				0.25	0.1	0.01	0.001	0.0001
BRI (quartile)								
Q2 vs. Q1	1.69 (1.11–2.58)	0.0186	0.51	**0.099/0.502**	0.247/**0.752**	0.783/0.971	0.973/0.997	0.997/1.000
Q3 vs. Q1	2.24 (1.53–3.27)	0.0002	0.11	**0.005/0.040**	**0.016/0.110**	**0.152/0.577**	0.644/0.932	0.948/0.993
Q4 vs. Q1	2.93 (1.94–4.44)	<0.0001	0.117	**0.003/0.008**	**0.008/0.023**	**0.078/0.208**	0.462/**0.726**	0.896/0.964

a Crude OR reported in Table 2.

b Weighted multivariate logistic regression models were used to calculate the association between BRI and infertility.

c Statistical power was calculated using the number of observations, crude OR, and *p*-values.

d FPRP < 0.2 and BFDP < 0.8 were considered noteworthy. The noteworthy results were highlighted in bold.

## Discussion

In women, obesity can affect fertility and reproduction in different ways, including interference with spontaneous ovulation, steroid metabolism and secretion, and insulin activity (25, 26). An increasing amount of research has indicated that visceral fat can have a more specific impact on fertility than subcutaneous fat (25–27). Therefore, exploring visceral fat markers for assessing the associations between obesity and infertility is valuable.

This study was the first to investigate the relationship between the BRI and female infertility using the NHANES data. We found that a higher BRI, a novel visceral fat-related anthropometric index, was associated with a higher risk of infertility among women aged 18 to 45 years. In the descriptive analyses, our results showed that the mean BRI values in the infertility group were significantly higher than those of the non-infertility group (Supplementary Table S1). These results suggest that the BRI has predictive value for differentiating fertility status. When we classified participants by the BRI quartiles, we observed a significant increase in both the mean WC and the percentage of infertility from Q1 to Q4 (Table 1). These results prompted us to further analyze the correlation between the BRI and infertility by developing weighted multivariate logistic regression models. As expected, the BRI as a categorical variable served as a more revealing independent risk factor for infertility than its continuous variable (Table 2).

In addition, further spline smoothing analysis revealed a non-linear positive relationship between the BRI and infertility (Figure 2). When the infection point was higher than 6.36, the OR decreased from 1.23 (1.15–1.32) to 1.13 (1.08–1.20; Table 3). These results suggest that the predictive value of visceral fat on infertility risk is more pronounced in the early stage of visceral fat accumulation. This finding aligns with a previous study indicating that early and consistent loss of intra-abdominal fat is associated with the resumption of ovulation (27). In addition, multiple studies have reported that the incidence of metabolic disorders, such as insulin resistance, hyperlipidemia, and glucose intolerance, increases with visceral fat accumulation (25, 26). These metabolic disorders are also important risk factors for infertility, which may also explain the slight decrease in OR values observed after the BRI value exceeded the infection point. Finally, using Bayesian statistics (FPRP and BFDP) to investigate the robustness of our results, we found significant findings for Q3 and Q4 at a prior probability of 0.01 in both tests. Notably, when assuming a prior probability of 0.001, the significant association of Q4 remained noteworthy for the BFDP test (Table 4). These findings further highlight the links between the BRI and infertility.

Recently, numerous studies have highlighted the use of novel indicators to more accurately predict the risk of infertility in women by measuring visceral fat, rather than relying on BMI, which fails to accurately distinguish between visceral and subcutaneous fat (28–32). For instance, Yang et al. reported a significant positive association between a body shape index (ABSI), a novel marker calculated using the BMI, WC, and height, as well as an increased risk of infertility in women (30). Similarly, the BRI, as a non-invasive indicator of visceral obesity, holds promise in identifying women at high risk for infertility. Additionally, we found that participants with infertility and higher BRI values were older and had lower education levels, lower PIR, earlier menarche, higher PID rates, and a higher prevalence of diabetes, hypertension, and dyslipidemia. Notably, a higher proportion of these participants identified as Mexican American or non-Hispanic Black (Table 1). These findings underscore the necessity of considering the complex interactions between the BRI and confounding factors when evaluating higher BRI as a risk factor for female infertility.

While our study elucidated a non-linear positive relationship between the BRI and infertility, enhancing our understanding of the intricate relationship between obesity and infertility, it is essential to acknowledge several limitations. First, as a cross-sectional study, it is difficult to make causal inferences between the BRI and infertility, as the association between them is complex. Additionally, our study has limitations in how infertility was assessed. The diagnosis relied on a reproductive health questionnaire completed by female participants, which may not accurately reflect the situation and does not include information on male infertility. Since infertility is influenced by factors from both partners, focusing solely on female data may lead to an underrepresentation of the true prevalence and overlook the full range of contributing factors. Future studies should incorporate data from male partners and adopt a more comprehensive approach to better understand the interplay between male and female factors, thereby improving the accuracy and generalizability of infertility research. Finally, since this study focused exclusively on the U.S. population, it is unclear whether our findings can be generalized to other countries or ethnicities and needs to be investigated.

## Conclusion

In summary, the findings of this study indicate that BRI has a non-linear positive association with the risk of female infertility. Given the detrimental effect of visceral fat on infertility in women, the BRI may serve as a valuable tool for the early identification of high-risk individuals.

## Data Availability

The datasets presented in this study can be found in online repositories. The names of the repository/repositories and accession number(s) can be found in the article/Supplementary material.
