# Supplementary material for: Association between body roundness index and female infertility: a cross-sectional study of NHANES 2013–2018
Source: Front Nutr. 2024 Dec 17;11:1509311. doi: 10.3389/fnut.2024.1509311 (PMC11685068; doi:10.3389/fnut.2024.1509311)
Supplement: Supplementary file 1 [file Table_1.DOCX]

| **Supplementary Table S1. Basic characteristics of participants^a^.** | | | |
| --- | --- | --- | --- |
| **Variables** | **Non-infertility**  **(N = 3,121)** | **Infertility**  **(N = 407)** | ***P*** |
| **Age (years)** | 30.90 (30.49-31.31) | 35.19 (34.26-36.11) | < 0.0001 |
| **Race (%)** |  |  | 0.0110 |
| Mexican American | 12.24 (9.66-15.39) | 10.34 (6.82-15.38) |  |
| Other Hispanic | 8.26 (6.79-10.03) | 5.45 (3.46-8.49) |  |
| Non-Hispanic White | 54.90 (50.11-59.61) | 63.51 (56.30-70.15) |  |
| Non-Hispanic Black | 13.59 (10.99-16.68) | 12.07 (9.11-15.81) |  |
| Other race | 11.01 (9.47-12.76) | 8.63 (6.22-11.85) |  |
| **Marital status (%)** |  |  | < 0.0001 |
| Married | 39.75 (37.03-42.53) | 64.46 (58.51-69.99) |  |
| Widowed | 0.30 (0.15-0.60) | 1.94 (0.59-6.19) |  |
| Divorced | 6.38 (5.19-7.82) | 5.89 (3.99-8.60) |  |
| Separated | 2.80 (2.23-3.51) | 3.34 (1.88-5.87) |  |
| Never married | 36.29 (33.63-39.03) | 13.24 (10.42-16.67) |  |
| Living with partner | 14.50 (13.01-16.13) | 11.14 (7.89-15.50) |  |
| **Education level (%)** |  |  | 0.2699 |
| Less than high school | 13.11 (11.41-15.02) | 10.21 (7.67-13.47) |  |
| High school or equivalent | 21.02 (18.63-23.64) | 19.62 (14.30-26.32) |  |
| College or above | 65.87 (62.26-69.30) | 70.17 (63.50-76.08) |  |
| **BMI (kg/cm^2^)** | 28.80 (28.33-29.27) | 31.56 (30.28-32.84) | 0.0004 |
| **PIR** | 2.57 (2.45-2.69) | 2.85 (2.62-3.08) | 0.0168 |
| **Blood cotinine (ng/mL)** | 40.19 (34.34-46.04) | 53.18 (40.10-66.27) | 0.0635 |
| **Drinking (%)** |  |  | 0.6803 |
| Non-drinker | 35.87 (32.61-39.26) | 37.90 (31.06-45.27) |  |
| 1-5 drinks/month | 33.12 (30.57-35.77) | 33.51 (27.97-39.54) |  |
| 5-10 drinks/month | 14.95 (12.90-17.26) | 11.76 (8.00-16.96) |  |
| 10^+^ drinks/month | 16.06 (13.55-18.95) | 16.83 (11.84-23.37) |  |
| **Height (cm)** | 162.48 (162.09-162.87) | 163.70 (162.80-164.59) | 0.0141 |
| **WC (cm)** | 94.28 (93.22-95.33) | 102.38 (99.71-105.06) | < 0.0001 |
| **Diabetes (%)** |  |  | 0.0012 |
| Yes | 2.52 (1.98-3.21) | 5.66 (3.72-8.54) |  |
| No | 97.48 (96.79-98.02) | 94.34 (91.46-96.28) |  |
| **Dyslipidemia (%)** |  |  | < 0.0001 |
| Yes | 14.55 (13.07-16.16) | 26.92 (21.86-32.66) |  |
| No | 85.45 (83.84-86.93) | 73.08 (67.34-78.14) |  |
| **Hypertension (%)** |  |  | < 0.0001 |
| Yes | 13.15 (11.70-14.75) | 23.39 (18.40-29.24) |  |
| No | 86.85 (85.25-88.30) | 76.61 (70.76-81.60) |  |
| **Menarche (years)** | 12.58 (12.51-12.65) | 12.49 (12.28-12.71) | 0.4192 |
| **PID (%)** |  |  | < 0.0001 |
| Yes | 3.79 (2.99-4.81) | 9.71 (6.58-14.10) |  |
| No | 96.21 (95.19-97.01) | 90.29 (85.90-93.42) |  |
| **Birth control pills (%)** |  |  | 0.0004 |
| Yes | 70.45 (67.91-72.87) | 80.45 (74.90-85.01) |  |
| No | 29.55 (27.13-32.09) | 19.55 (14.99-25.10) |  |
| **Female hormones (%)** |  |  | 0.0026 |
| Yes | 3.57 (2.61-4.86) | 9.29 (5.13-16.26) |  |
| No | 96.43 (95.14-97.39) | 90.71 (83.74-94.87) |  |
| **BRI** | 5.24 (5.08-5.40) | 6.30 (5.87-6.74) | < 0.0001 |

**Abbreviations:** BMI, body mass index; PIR, poverty impact ratio; WC, waist circumference; PID,

pelvic infection/pelvic inflammatory disease; BRI, body roundness index.

^a^Percentage estimates were nationally representative through the use of survey weights.
